# mTOR Modulation of *I_Kr_* through hERG1b-Dependent Mechanisms in Lipotoxic Heart

**DOI:** 10.3390/ijms23158061

**Published:** 2022-07-22

**Authors:** Kelly A. Aromolaran, Jenny Do, Joyce Bernardi, Ademuyiwa S. Aromolaran

**Affiliations:** 1 Nora Eccles Harrison Cardiovascular Research and Training Institute (CVRTI), University of Utah School of Medicine, Salt Lake City, UT 84112, USA; kelly.aromolaran@utah.edu; 2 Masonic Medical Research Institute, Utica, NY 13501, USA; jennydo1536@gmail.com (J.D.); jbernardi@mmri.edu (J.B.); 3 Department of Surgery, Division of Cardiothoracic Surgery, University of Utah School of Medicine, Salt Lake City, UT 84112, USA

**Keywords:** lipotoxicity, hERG1a/1b, *I_Kr_*, guinea pig, atrial myocytes, mTOR

## Abstract

In the atria, the rapid delayed rectifier channel (*I_Kr_*) is a critical contributor to repolarization. In lipotoxic atria, increased activity of the serine/threonine mammalian target of rapamycin (mTOR) may remodel *I_Kr_* and predispose patients to arrhythmias. To investigate whether mTOR produced defects in *I_Kr_* channel function (protein expression and gating mechanisms), electrophysiology and biochemical assays in HEK293 cells stably expressing hERG1a/1b, and adult guinea pig atrial myocytes were used. Feeding with the saturated fatty acid palmitic acid high-fat diet (HFD) was used to induce lipotoxicity. Lipotoxicity-challenged HEK293 cells displayed an increased density of hERG1a/1b currents due to a targeted and significant increase in hERG1b protein expression. Furthermore, lipotoxicity significantly slowed the hERG1a/1b inactivation kinetics, while the activation and deactivation remained essentially unchanged. mTOR complex 1 (mTORC1) inhibition with rapamycin (RAP) reversed the increase in hERG1a/1b density and inactivation. Compared to lipotoxic myocytes, RAP-treated cells displayed action potential durations (APDs) and *I_Kr_* densities similar to those of controls. HFD feeding triggered arrhythmogenic changes (increased the *I_Kr_* density and shortened the APD) in the atria, but this was not observed in low-fat-fed controls. The data are the first to show the modulation of *I_Kr_* by mTORC1, possibly through the remodeling of hERG1b, in lipotoxic atrial myocytes. These results offer mechanistic insights with implications for targeted therapeutic options for the therapy of acquired supraventricular arrhythmias in obesity and associated pathologies.

## 1. Introduction

Atrial fibrillation (AF) is the most common arrhythmia in humans [1,2] and a leading cause of morbidity and mortality worldwide [2,3]. AF’s pathogenesis results from adverse atrial electrical remodeling due to defects in the functional expression of major ion channels [4,5]. Obesity is a growing epidemic and a key contributor to the increasing prevalence of AF [1,6]. Accordingly, obesity may have a direct impact on the electrical activity of the atria [7]. Obesity-related arrhythmias are associated with the abnormal cardiac accumulation of free fatty acids [8]. Preventing such cardiac lipotoxic effects is a potential avenue for novel therapeutic interventions in obese patients with AF; however, the molecular mechanisms of lipotoxicity are poorly understood.

In the human heart, pore-forming human ether-á-go-go related gene (hERG) subunits (1a/1b) form the rapidly activating component (*I_Kr_*) of the delayed rectifier K current (*I_K_*). We previously demonstrated that pathological remodeling of *I_Kr_* can be induced by lipotoxicity [5]. This remodeling results in increased repolarizing current amplitudes and associated cellular proarrhythmic mechanisms including shortened action potential durations (APDs), early afterdepolarizations, and an increased incidence of spontaneous beats [5]. Moreover, there have been reports showing an important contribution of K currents to AF pathogenesis [5,9]. Collectively, this suggests that understanding the modulation of K channels during lipotoxicity may be essential for elucidating the pathogenesis of AF. This objective can be pursued by exploring whether and how key lipotoxicity signaling pathways, triggered in response to nutritional excess, affect atrial electrical activity.

Lipotoxicity is associated with the activation of mTOR [10]. mTOR is a serine–threonine kinase that plays a central role in integrating the cell’s response to nutritional status [11,12], serving as a key signaling intermediate to regulate cellular functions, including protein synthesis [12]. The mTOR pathway is activated by phosphatidylinositide 3-kinase (PI3K)–Akt, is inhibited by 5’ adenosine monophosphate-activated protein kinase (AMPK), and leads to cardiac hypertrophy [13].

mTOR contributes to the upregulation of neuronal voltage-gated K channel currents [14,15], the acceleration of channel activation, and the slowing of channel inactivation [14]. In a genome-wide association study, Furukawa and co-workers recently established that the mTOR pathway was associated with the pathogenesis of AF [16], while Liu and co-workers demonstrated that mTOR expression was increased in both an acute and chronic beagle AF model [17]. Thus, the mTOR signaling pathway looms as a strong candidate for the modulation of *I_Kr_* function and the associated cellular manifestations of supraventricular arrhythmias in the lipotoxic heart. Here, we unravel a novel lipotoxicity-associated channelopathy (possibly due to dysfunction in the hERG1b subunits) as a risk factor for AF, whereby the mTOR-mediated upregulation of *I_Kr_* in lipotoxic atria underlies APD shortening, which promotes atrial arrhythmogenesis.

## 2. Results

### 2.1. mTOR Inhibition Prevented Increases in hERG1a/1b Currents in Lipotoxic HEK293 Cells

**Figure 1:**
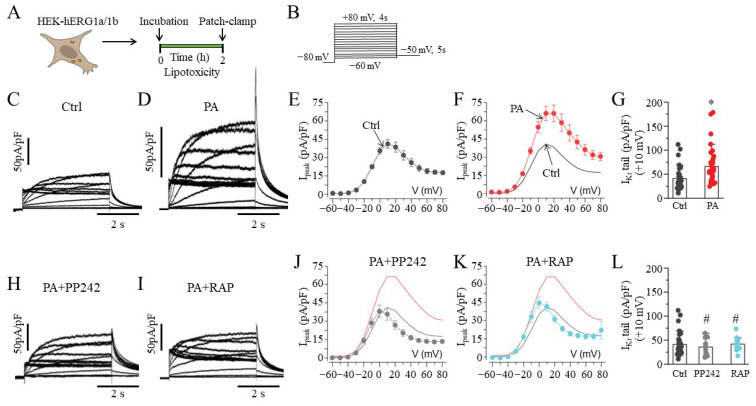
**Electrophysiology of hERG1a/1b currents in lipotoxic HEK293 cells.** (**A**) Experimental protocol: Macroscopic hERG1a/1b currents measured in HEK293 cells under control conditions and after pre-exposure to lipotoxicity for 2 h. (**B**) Voltage protocols used for evoking hERG1a/1b currents. Exemplar hERG1a/1b currents measured in control (**C**) and lipotoxic HEK293 cells (**D**). (**E**–**G**), Population *I_peak_*–*V* curves for hERG1a/1b currents and averaged data measured at +10 mV under the indicated conditions. *Arrows* indicate pooled 1a/1b peak current in control and lipotoxic cells. (**H**–**L**), Representative traces, population *I_peak_–V* curves, and averaged data at +10 mV for currents measured in lipotoxic cells pre-exposed to PP242 (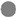, and *grey line*) and RAP (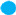, and *cyan line*), with the same format as (**C**–**G**). The *I*_peak_–*V* curves for hERG1a/1b currents measured in control cells (*black line*) and in cells pre-treated with lipotoxicity (*red line*) are shown for comparison. Data are shown as the mean ±S.E.M. (* statistical significance at *p* < 0.05; ^#^ statistical non-significance at *p* > 0.05).

We evaluated the impact of the saturated free fatty acid PA (BSA-palmitate, 0.5 mM) on the current density of hERG1a/1b. Whole-cell patch-clamp experiments were conducted at room temperature (23–25 °C) in lipotoxic HEK-hERG1a/1b cells (Figure 1A), using the protocol illustrated in Figure 1B. Compared to basal hERG1a/1b currents (Figure 1C), lipotoxic cells (Figure 1D) displayed increased current density (Figure 1E,F). At +10 mV, the hERG1a/1b current density was increased by 60.9% (from 41.2 ± 3.54 pA/pF, *n* = 42, to 66.3 ± 5.95 pA/pF, *n* = 37; * *p* < 0.05; Figure 1G).

We next examined whether the current density of hERG1a/1b currents in lipotoxic cells was dependent on mTOR activity by pretreating (≥30 min) cells with PP242, a selective and ATP-competitive mTOR inhibitor (1 μM, Figure 1H), or rapamycin (RAP, 250 nM; Figure 1I) [18]. Cells were subsequently challenged with BSA-palmitate for an additional 2 h in the continued presence of the mTOR inhibitors. PP242 and RAP prevented the increases in peak density observed in lipotoxic cells. At +10 mV, the hERG1a/1b peak current density was reduced from 66.3 ± 5.95 pA/pF (control, *n* = 37) to 35.7 ± 4.92 pA/pF (PP242, *n* = 12; Figure 1J) and 41.8 ± 3.75 pA/pF (RAP, *n* = 13; Figure 1K), comparable to basal currents (Figure 1L).

### 2.2. Lipotoxicity-Induced Increases in hERG1a/1b Involve PI3K/Akt and AMPK Pathways

**Figure 2:**
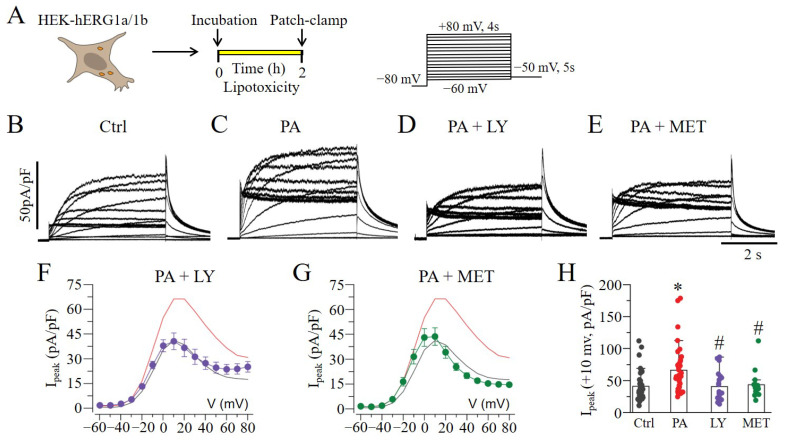
**The effect of mTOR signaling on hERG1a/1b currents.** (**A**) Experimental protocol: Macroscopic hERG1a/1b currents measured in HEK293 cells under control conditions and 2 h after lipotoxicity exposure, using the illustrated voltage protocol. (**B**–**E**) Exemplar traces of currents measured in control and lipotoxic cells (PA) in the absence and presence of the PI3K–Akt inhibitor LY and AMPK activator MET. (**F**–**H**), Pooled *I_peak_*–*V* curves, and averaged peak current at +10 mV for 1a/1b measured in lipotoxic cells in the presence of LY (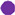, and *purple line*) and MET (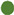, and *green line*). The *I*_peak_–*V* curves for hERG1a/1b currents measured in control cells (*black line*) and in cells pre-treated with lipotoxicity (*red line*) are shown for comparison. (* statistical significance at *p* < 0.05; ^#^ statistical non-significance at *p* > 0.05).

Previous observations have shown that the mTOR pathway is activated by PI3K–Akt and inhibited by AMPK [13]. Next, we investigated whether the pharmacological blockade of PI3K–Akt or activation of AMPK would affect lipotoxicity-induced increases in the hERG1a/1b current density in HEK293 cells (Figure 2A). Figure 2B shows typical hERG1a/1b currents measured under basal conditions. Exposure to lipotoxicity (BSA-palmitate at 0.5 mM for 2 h) significantly increased the hERG1a/1b density (Figure 2C), while the pre-exposure (≥30 min) of cells to a PI3K–Akt inhibitor, LY294002 (20 μM; Figure 2D), or an AMPK activator, metformin (MET, 5 mM; Figure 2E), completely reversed this effect (Figure 2F–H). Similar to the averaged control value (41.2 ± 3.54 pA/pF, *n* = 42) measured at +10 mV, the hERG1a/1b current densities were 40.6 ± 5.03 pA/pF (LY294002, *n* = 21) and 43.6 ± 5.28 pA/pF (MET, *n* = 16) (Figure 2H).

### 2.3. Lipotoxicity Upregulates hERG1a/1b Current Density by Promoting Translation of hERG1b

**Figure 3:**
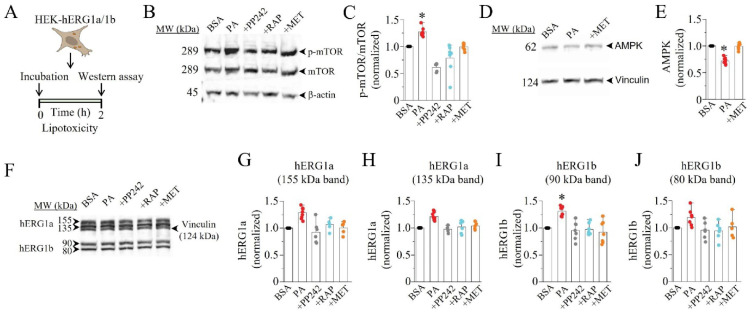
**Western blot assay revealed modulation of mTOR, AMPK, and hERG1a/1b expression in lipotoxic HEK293 cells.** (**A**) Experimental protocol: mTOR, AMPK, and hERG1a/1b expression in HEK293 cells under control conditions and after pre-exposure to lipotoxicity for 2 h was measured. (**B**) Western blot assays show the levels of activated mTOR (p-mTOR) induced by lipotoxicity in the absence or presence of mTOR inhibitors (PP242 and RAP), or AMPK activator (MET), compared to in control cells. (**C**) Quantification of the Western blot and statistical analysis showing the effects of PP242, RAP, and MET on the levels of p-mTOR. (**D**,**E**) Western blot and quantification/statistical analysis of AMPK expression in control and lipotoxic cells in the presence or absence of MET. (**F**–**J**) Western blot and quantification/statistical analysis showing the levels of hERG1a and 1b expression in the absence and presence of PP242, RAP, and MET in control and lipotoxic cells. (* statistical significance at *p* < 0.05).

We hypothesized that the PA-induced increase in hERG1a/1b current density might be due to an mTOR-dependent upregulation of hERG1a and 1b expression. To test this, we first assessed whether lipotoxicity affected mTOR and AMPK protein levels using Western blot analysis (Figure 3A). Compared to non-lipotoxic cells (Figure 3B), we found that lipotoxicity significantly increased phosphorylated mTOR (P-mTOR) protein, by 27% (Figure 3C, * *p* < 0.05, *n* = 7). We further found that pre-exposure (≥30 min) to PP242, RAP, or MET prevented the upregulation of p-mTOR (Figure 3C). A similar picture emerged with AMPK (Figure 3D). Thus, compared to controls, lipotoxic cells displayed a significant depression of AMPK protein levels (28%, Figure 3E; * *p* < 0.05, *n* = 9), and this was prevented by pre-exposure to the AMPK activator MET (Figure 3E).

Next, we assessed whether lipotoxicity affected hERG protein. We examined the protein levels of hERG1a and 1b in control cells; lipotoxic cells; and lipotoxic cells pre-exposed to PP242, RAP, and MET. As illustrated in Figure 3F, the untreated control cells displayed channel-specific hERG1a (155 and 135 kDa) and hERG1b (90 and 80 kDa) bands. Compared to the control, the hERG1a mature and immature band intensities were increased by 29% (*p* > 0.05, *n* = 9; Figure 3G) and 22% (*p* > 0.05, *n* = 9; Figure 3H) in lipotoxic cells, respectively. The pre-exposure (≥30 min) to PP242, RAP, or MET prevented the effects of lipotoxicity on hERG1a expression. A similar picture also emerged with the hERG1b mature (Figure 3I) and immature bands (Figure 3J). In contrast to that for hERG1a, lipotoxicity exerted a more pronounced effect on the hERG1b mature band (increased by 31%, *n* = 8, * *p* < 0.05; Figure 3I). The data demonstrate that lipotoxicity increases hERG1a/1b density by upregulating hERG1b expression.

**Figure 4:**
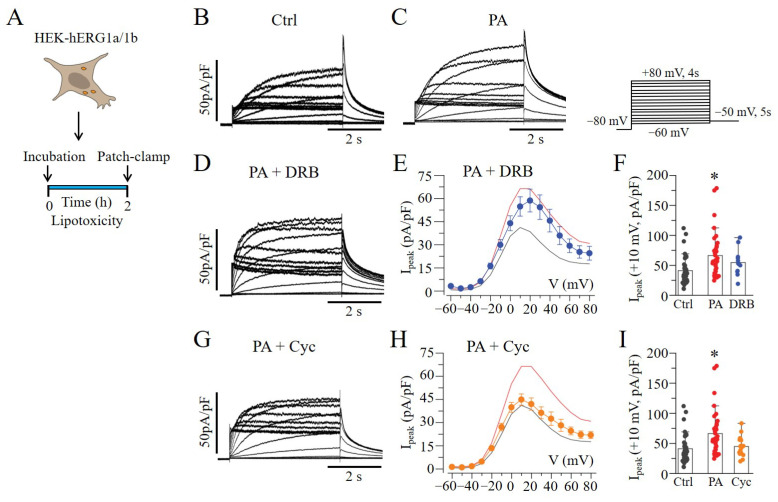
**Protein translation contributes to lipotoxicity-induced increase in hERG1a/1b.** (**A**) Experimental protocol: macroscopic hERG1a/1b currents were measured in HEK293 cells under control conditions (**B**) and after pre-exposure to lipotoxicity (**C**) for 2 h, using the illustrated voltage protocol. (**D**–**I**) Exemplar traces, pooled *I_peak_*–*V* curves, and averaged current amplitude measured at +10 mV under the effects of DRB (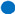, and *blue line*) and Cyc (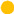, and *orange line*). The *I*_peak_–*V* curves for hERG1a/1b currents measured in control cells (*black line* in (**E**,**H**)) and in cells pre-treated with lipotoxicity (*red line in* (**E**,**H**)) are shown for comparison. (* statistical significance at *p* < 0.05).

Next, we hypothesized that changes in hERG1a and hERG1b gene expression in response to lipotoxicity may occur at the transcriptional level. To test this, we assessed the effects of lipotoxicity on hERG1a/1b current densities (Figure 4A), pre-treated with or without the transcription inhibitor 5,6-dichloro-1-β-D-ribofuranosylbenzimidazole (DRB, 10 μM) [15,19]. Figure 4B shows hERG1a/1b currents measured in non-lipotoxic cells. Compared to in lipotoxic cells (Figure 4C), pre-exposure (≥30 min) to DRB failed to prevent the increases in hERG1a/1b density (Figure 4D–F). Our data suggest that transcription is not required for the lipotoxicity-dependent increase in hERG1a/1b expression and current density in HEK293 cells.

We then examined whether lipotoxicity upregulated the levels of hERG by increasing the translational efficiency for hERG1a/1b using the translation inhibitor cycloheximide (Cyc, 10 μM). As illustrated in Figure 4G, pre-incubation (≥30 min) with Cyc completely reversed the effects of lipotoxicity on hERG1a/1b density (Figure 4H,I). Together, our data suggest that lipotoxicity acts at the translational level to enhance the expression of the hERG channel protein and hERG1a/1b density.

### 2.4. mTOR Inhibition Slowed hERG Channel Inactivation in HEK293 Cells

**Figure 5:**
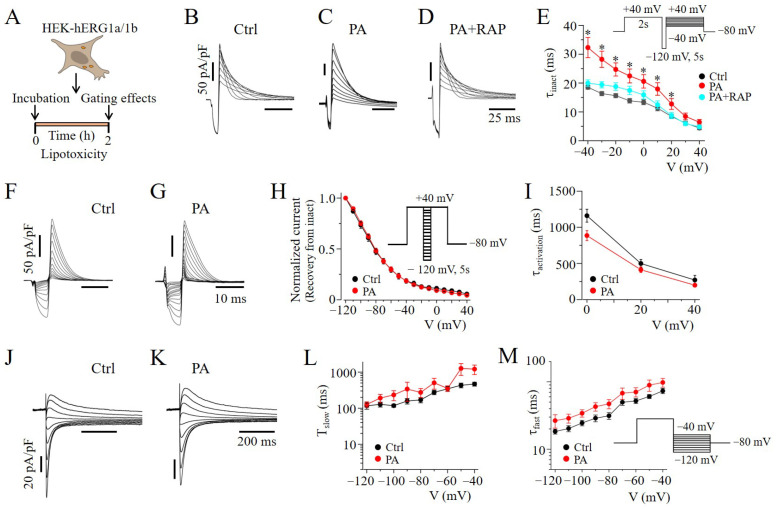
**Rapamycin affects hERG1a/1b channel inactivation in lipotoxic HEK293 cells.** (**A**) Experimental protocol: hERG1a/1b channel gating mechanisms were determined in HEK293 cells under control conditions, and after pre-exposure to lipotoxicity for 2 h, using the illustrated voltage protocols. Exemplar current traces (**B**–**D**) and a graph of τ_inactivation_ (**E**) measured under the indicated conditions. τ_inactivation_ was determined by fitting single exponential functions to the decaying phase of currents at voltages ranging from −40 to +40 mV. Pre-exposure to RAP prevented the slowing of τ_inactivation_ due to lipotoxicity. The hERG1a/1b channels’ recovery from the voltage-dependent inactivation (**F**–**H**), activation (**I**), and deactivation (**J**–**M**) kinetics were essentially unaltered under lipotoxic conditions. (* statistical significance at *p* < 0.05).

To further determine the mechanism underlying the mTOR-dependent regulation of hERG1a/1b density, we assessed the impact of RAP on hERG1a/1b gating mechanisms in lipotoxic cells (Figure 5A). Compared to controls (Figure 5B), cells exposed to lipotoxicity had a significant increase in the inactivation time constant (τ_inactivation_). At +10 mV, τ_inactivation_ was increased from 11.2 ± 0.91 ms (*n* = 7) to 17.9 ± 2.16 ms (*n* = 18, * *p* < 0.05; Figure 5B,C,E) when cells were exposed to PA for 2 h prior to recording. Pre-exposing (≥30 min) lipotoxic cells to RAP generated a τ_inactivation_ that was similar to that for untreated cells (11.2 ± 0.91 vs. 12.4 ± 1.37 ms, *n* = 7, *p* > 0.05; Figure 5D,E), suggesting that mTOR contributes to the effects of lipotoxicity on hERG channel inactivation. By contrast, lipotoxicity had no effects on the voltage-dependent recovery from inactivation (Figure 5F–H), rise time of activation (τ_activation_*,* Figure 5I), or deactivation kinetics (τ_slow_ and τ_fast,_ Figure 5J–M).

### 2.5. mTOR Activation Increased I_Kr_ and Shortened APDs in Lipotoxic Atrial Myocytes

**Figure 6:**
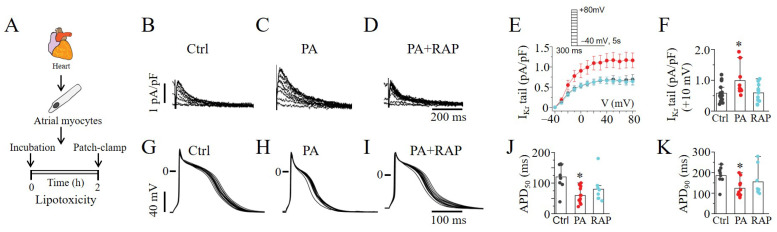
**Electrophysiology of *I_Kr_* and action potentials in guinea pig atrial cardiomyocytes.** (**A**) Experimental protocol: *I_Kr_* tail currents and AP were measured in freshly isolated atrial myocytes from adult guinea pig heart under control conditions and after pre-exposure to lipotoxicity for 2 h. Exemplar *I_Kr_* tail current traces measured in control cells (**B**) and lipotoxic cells in the absence (**C**) and presence of RAP (**D**). (**E**) Population *I–V* curves for *I_Kr_* tail currents measured in control cells (*black symbol and line*), lipotoxic cells (*red symbol and line*) and in lipotoxic cells pre-treated with RAP (*cyan symbol and line*). (**F**) *I_Kr_* tail current amplitude measured at +10 mV under the indicated conditions. Representative AP traces measured in control cells (**G**) and after pre-exposure to lipotoxicity (2 h, **H**) and lipotoxicity+RAP (**I**). Statistical analysis of APD_50_ (**J**) and APD_90_ (**K**) revealed that RAP partially rescued lipotoxicity-induced shortening of APD in atrial myocytes. Data were generated from cardiomyocytes from three guinea pigs. (* statistical significance at *p* < 0.05).

mTOR’s effects were examined by whole-cell patch-clamp experiments in lipotoxic atrial cardiomyocytes (Figure 6A). Similar to the observations in HEK293 cells, lipotoxicity dramatically increased atrial *I_Kr_* tail currents (Figure 6B,C). In the presence of RAP (Figure 6D), the increase was significantly reduced from 0.99 ± 0.17 pA/pF (*n* = 7) to 0.59 ± 0.08 pA/pF (*n* = 12, * *p* < 0.05; Figure 6E,F), similar to the control (0.58 ± 0.06 pA/pF, *n* = 17). Next, we determined the effects of mTOR inhibition on the APD (Figure 6G), measured in lipotoxic atrial myocytes (Figure 6H). Using a whole-cell current clamp, we demonstrated APD shortening at both 50% (APD_50_) and 90% (APD_90_) repolarization in lipotoxic myocytes compared to control (Figure 6G,H). However, the pre-exposure (≥30 min) of myocytes to RAP (Figure 6I) only partially reversed the APD_50_ (Figure 6J) and APD_90_ (Figure 6K). These data suggest that mTOR selectively modulates *I_Kr_* in the lipotoxic heart and further support a role for the remodeling of the function of other major atrial currents, due to lipotoxicity independent of modulation by mTOR signaling.

**Figure 7:**
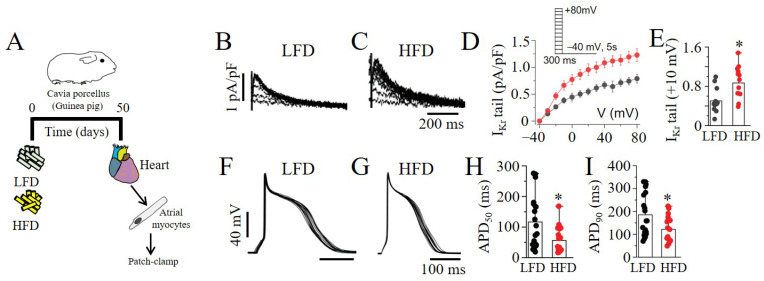
**High-fat-diet feeding upregulated *I_Kr_* and shortened action potential duration in adult guinea pig atria.** (**A**) Experimental protocol: *I_Kr_* tail current density and APs were measured in guinea pigs fed an LFD or HFD for 50 days. (**B**–**E**) Exemplar *I_Kr_* tail current traces, population *I_peak_*–*V,* and averaged current amplitude at +10 mV measured in freshly isolated atrial myocytes. Pooled *I–V* curves (in **D**) represent *I_Kr_* tail current density measured in LFD-fed control myocytes (*black symbol and line*) and HFD-challenged myocytes (*red symbol and line*). (**F**–**I**) Current-clamp analysis of AP morphology in atrial myocytes from LFD- and HFD-fed guinea pigs. Electrophysiology data were generated from cardiomyocytes from three guinea pigs per diet group. (* statistical significance at *p* < 0.05).

Electrophysiology assays were used to investigate the impact of the LFD and HFD feeding of guinea pigs on atrial myocyte electrical activity (Figure 7A). Using whole-cell and current-clamp experiments, we demonstrated increased *I_Kr_* tail current density (Figure 7B–E) and APD (APD_50_ and APD_90_; Figure 7F–I) shortening in the myocytes from the HFD- compared to LFD-exposed guinea pigs. Together, our data imply that HFD feeding triggers dramatic and potentially arrhythmogenic changes in *I_Kr_* properties and AP morphology in the atria, possibly through mTOR activation.

## 3. Discussion

Obesity is a key contributor to the increasing prevalence of AF. Obesity-associated lipotoxicity is an independent risk factor for arrhythmias [8]. Increased *I_Kr_* density, a critical determinant of cardiac repolarization, can be induced by systemic lipotoxicity [20], elevating the risk for supraventricular arrhythmias [5]; however, the cellular proarrhythmic mechanisms are poorly understood. The goal of this study was to determine whether a repair mechanism preventing the adverse remodeling of *I_Kr_* in lipotoxicity existed. Here, we show that the selective inhibition of mTORC1 with RAP normalized lipotoxicity-induced increases in *I_Kr_* density in heterologous cells and atrial myocytes resulting in the prolongation of APD. Our data further demonstrate that the ability of mTORC1 to augment *I_Kr_* is due to defects in protein translation and channel gating. Thus, elevated mTORC1 expression and increased *I_Kr_* may contribute to early repolarization, leading to the increased susceptibility to supraventricular arrhythmias reported in obese patients [21].

While there is evidence that mTOR dysregulation in pathological conditions contributes to heart disease [13], to our knowledge, there have been no studies on the modulation of hERG/*I_Kr_* by mTOR. In support of our study, Tyan et al. [14] showed that mTOR upregulated voltage-gated K currents (Kv1.3 and Kv1.5) in *Xenopus oocytes*. Yao et al. [15] also demonstrated that the blockade of mTOR with RAP prevented neureglin-1-mediated increases in transient outward K currents in rat cerebellar granule neurons. Our data are consistent with the modulation of cardiac hERG/*I_Kr_* channels by the mTOR signaling complex.

mTORC1 is involved in the regulation of protein synthesis and energy metabolism, whereas mTORC2 plays an important role in regulating actin dynamics [22]. We used RAP (which specifically blocks mTORC1 [23]) and PP242 (which inhibits mTOR’s catalytic activity) to perturb both mTORC1 and mTORC2 [24]. Previous studies have shown that RAP prevented the neureglin-1/ErB4-mediated increase in Kv4.2 protein expression and the associated transient outward K current (*I_A_*) in rat cerebellar granule neurons [15]. Similarly, Nguyen et al. [25] demonstrated that RAP prevented the increases in Kv1.1 protein expression in a neuronal subset-specific *Pten*-knockout mouse model. Our present data are in agreement with these observations. Importantly, our studies highlight a key role for modulation by the mTORC1-dependent hERG1b translation of *I_Kr_* in lipotoxicity.

We further discovered that BSA-palmitate-challenged HEK293 cells display increased protein expression of mTOR, consistent with the activation of the hERG1b protein translation pathway in the context of lipotoxicity. Additionally, the reversal of the hERG1a/1b electrophysiological phenotype and protein levels after the inhibition of PI3K–Akt or the activation of AMPK suggest that translational changes in the protein levels of hERG1a/1b caused by lipotoxicity could contribute to alterations in the atrial electrophysiological phenotype. Thus, the PI3K/Akt and AMPK pathways could act through mTOR and trigger dramatic and potentially arrhythmogenic changes in *I_Kr_* and APD, ultimately elevating the risk for atrial arrhythmogenesis [26,27], particularly in obese patients [26].

There are currently no reports on mTOR’s effects on *I_Kr_* channel gating. To our knowledge, there has only been one other study on the functional impact of mTOR on voltage-gated Kv1.3 channels. Tyan et al. reported that mTOR accelerated Kv1.3 channels’ activation and slowed their inactivation in *Xenopus oocytes*. In agreement, our analyses demonstrated that mTOR only slowed the inactivation of hERG1a/1b channel complexes under lipotoxicity. By providing the first measurements of mTOR’s effects on the biophysical properties of hERG1a/1b, we provide a new insight that the facilitatory effect of lipotoxicity on *I_Kr_* is due to defective hERG1b protein translation and hERG1a/1b channel gating.

The present study is the first to demonstrate that pathologically elevated mTOR levels can adversely modulate *I_Kr_* in the atria. Our findings are consistent with the notion that changes in mTOR in individuals with systemic inflammation related to HFD may lead to enhanced *I_Kr_*, with implications for cardiac repolarization [8]. Our results suggest that specific inhibitors of mTORC1 or cellular mediators that reduce hERG1b protein synthesis or channel opening to normalize *I_Kr_* may suppress the vulnerability to arrhythmogenic changes in atrial APD. By providing the first measurements of the effect of mTOR on hERG1a/1b, we reveal that *I_Kr_* is sensitive to pathological changes in the mTORC1/S6K1 pathway (an established protein translation pathway) in cardiac and systemic lipotoxicity. Collectively, our findings open new directions for determining the contribution of other downstream complex effectors (PI3K/Akt and AMPK) [28] in the lipotoxicity-mediated modulation of *I_Kr_*.

### Limitations of the Study

This study focused entirely on elucidating the involvement of mTOR signaling in the lipotoxicity-induced adverse remodeling of atrial electrical activity and the hERG/*I_Kr_* channel, to better define the cellular proarrhythmic mechanisms involved. A role for the transcriptional regulation of hERG warrants further investigation, as our experimental design may have limited the sensitivity of hERG/*I_Kr_* to transcriptional modulation. Another limitation is that we did not investigate phospho -AMPK/AMPK in HEK293 cells or mTOR expression in the diet-challenged guinea pigs. However, lipotoxicity is associated with the activation of mTOR [10] and a reduction in phospho-AMPK levels [29,30], and, therefore, likely mediates the effects reported in our study. Therefore, the data will require confirmation using PCR and Western Blot assays in future studies. Other mechanisms such as a reduced intracellular calcium transient amplitude [31,32,33] may have also contributed to the adverse changes in APD. Therefore, studies that distinguish between the functional expression of atrial calcium-handling proteins, with implications for reduced L-type Ca currents, are likely to provide critical insights for devising targeted therapies in obese patients. Work to advance these approaches is currently being conducted in our laboratory.

## 4. Materials and Methods

### 4.1. Cell Culture

Low-passage HEK293 cells stably expressing hERG1a/1b channels, a gift from Dr. Gail Robertson (University of Wisconsin-Madison), were cultured as previously described [34,35]. Briefly, the cells were maintained in DMEM supplemented with 10% FBS, and 100 g mL^−1^ penicillin–streptomycin at 37 °C for selection. hERG1a and hERG1b expression was maintained by adding 5 µg/mL G418 (G418, Gibco; Grand Island, NY, USA) and 0.25 µg/mL puromycin, respectively, every other day during medium changes. To induce hERG1b protein expression, doxycycline at a final concentration of 100 ng/mL was directly added to the medium 24 h before the experiments.

### 4.2. Guinea Pig Atrial Myocyte Isolation

Adult male and female Hartley guinea pigs were deeply anesthetized with isoflurane in accordance with the guidelines of the Declaration of Helsinki and as approved by the Institutional Review Board (or Ethics Committee) of the Masonic Medical Research Institute Use Committees, conforming to NIH guidelines. The primary myocyte isolation procedures were previously described [5]. Briefly, adult male and female Hartley guinea pig hearts were excised, and Langendorff perfused with Tyrode solution containing (in mM) 118 NaCl, 4.8 KCl, 1 CaCl_2_, 10 glucose, 1.25 MgSO_4_, and 1.25 K_2_HPO_4_ (pH = 7.4) for 5 min. Atrial myocytes were isolated by enzymatic digestion in Ca^2+^-free Tyrode solution containing collagenase B (final concentration, 0.6 mg/mL; Boehringer Mannheim, Indianapolis, IN, USA) for an additional 6 min. The heart was subsequently perfused with high-K solution containing (in mM): 70 KOH, 50 L-glutamic acid (potassium salt), 40 KCl, 10 taurine, 2 MgCl_2_, 10 glucose, 10 HEPES, 5 EGTA, and 1% albumin (pH 7.4, with KOH) for 10 min. The digested heart tissue was placed in fresh high-*K* solution, minced into smaller pieces, and triturated several times to dissociate the cells. The cell suspension was filtered through a mesh strainer and allowed to settle for 15–20 min. The pellet was resuspended in 10% M199 medium and plated on laminin-coated coverslips. The cells were patched 6–8 h after plating.

### 4.3. Preparation of Bovine Serum Albumin (BSA)-Conjugated FFA Solutions

Palmitic acid (PA) stock solution was prepared as previously described [20]. Fatty-acid-free bovine serum albumin (BSA, Roche) (20%) was dissolved in Dulbecco Phosphate Buffered Saline (DPBS) and then filtered to sterilize it. PA (Sigma-Aldrich, St. Louis, MO, USA) was dissolved in ethanol to generate a 0.2 M fatty acid (FA) stock solution. The BSA (20%) and FA (0.2 M) were mixed in a 20:1 volumetric ratio. FA stock solutions (~10 mM) were directly added to M199 culture medium or Tyrode’s solution to a final concentration of 0.5 mM. The vehicle-control solution was prepared with BSA, ethanol, and DPBS.

### 4.4. Low-Fat-Diet and High-Fat-Diet (Palmitic-Acid Diet) Feeding in Guinea Pigs

Guinea pigs (male/female; 200–250 g) were purchased from Charles River Laboratories (Wilmington, MA, USA). The control guinea pigs were fed, ad libitum, a low-fat diet (LFD, Research Diets Inc., New Brunswick, NJ, USA) containing (in kcal%): 10 fat, 70 carbohydrates, 20 protein, and 2300 corn starch. The PA-diet group was fed a diet (in which most of the soybean was replaced with 315 kcal% palm oil) containing 10% of its kilocalories from fat, 70% from carbohydrates, and 20% from protein. The PA-rich diet contained saturated and unsaturated free fatty acids (FFAs), which provided 48.4 and 36.8% of the fat-derived calories. The guinea pigs were fed an LFD or palm-oil-rich diet for a duration of 50 days (~7 weeks).

### 4.5. Electrophysiology

Whole-cell membrane currents were recorded in HEK293 cells and atrial cardiomyocytes using an Axopatch-200B amplifier (Axon Instruments, Inc., Burlingame, CA, USA). A coverslip with adherent HEK293 cells or myocytes was placed on the glass bottom of a recording chamber (0.7–1 mL in volume) mounted on the stage of an inverted microscope (Eclipse Ti-U microscope or Diaphot, Nikon). Micropipettes were made from 1.5 mm thin-walled glass and fire-polished. The internal solution contained (in mM): 133 KCl, 0.4 GTP, 10 EGTA, 1 MgSO_4_, 5 K_2_ATP (added on the day of experimentation), 0.5 CaCl_2_, and 10 HEPES (pH 7.2). The external solution contained (in mM): 147 NaCl, 4 KCl, 2 CaCl_2_, and 10 HEPES (pH 7.4). The pipette resistance was typically 1.5–2 MΩ when it was filled with internal solution. Pooled *I_peak_–V* curves for hERG1a/1b currents were generated from a family of step depolarizations (−60 to +80 mV in 10 mV steps for 4 s from a holding potential of −80 mV), followed by a step of repolarizing to −50 mV for 5 s to obtain tail currents. The currents were sampled at 20 kHz and filtered at 5 or 10 kHz. Traces were acquired at a repetition interval of 10 s. The cell capacitance or cell size (in pF) was compensated for and measured using the built-in compensation unit of the amplifier. To determine the time course of inactivation (τ_inactivation_) or time (10–90%) at which the peak occurred (activation time course), the data were fitted with a single exponential function. The time constants of the rate of current deactivation (τ_slow_ and τ_fast_), measured at −120 and −60 mV, were fitted to a double exponential function of the form y = y0 + A1 x exp(−x/τ_fast_) + A2 x exp(−x/τ_slow_), where t is the time. To determine the voltage-dependence recovery from inactivation, the peak current amplitude after the return to +40 mV was normalized and plotted against the potential of the hyperpolarizing step. The data were fitted to a built-in Boltzmann function in the Origin software.

The *I_Kr_* tail currents in atrial cardiomyocytes were evoked using a short 300 ms depolarizing pulse from a holding potential of −40 mV, and test pulses were applied at various voltages from −40 to +80 mV in 10 mV increments before returning to −50 mV for tail current recording. Action potential waveforms were continuously recorded from the atrial myocytes in current-clamp mode by passing depolarizing currents for 20 ms at a subthreshold (1.5 X) intensity in 10 s intervals for ~2 min. All the experiments were performed at room temperature (20–25 °C). Unless or otherwise stated, HEK293 cells or atrial myocytes were pretreated (≥30 min) with test drugs and subsequently exposed to BSA-palmitate (0.5 mM to induce lipotoxicity) for an additional 2 h in the continued presence of the specified test drug. Electrophysiological data were generated from 3–5 separate passages of HEK293 cells and 3 guinea pigs.

### 4.6. Western Blot Analysis

Whole cell lysates from HEK293 stably expressing hERG1a/1b channels were used for Western blot analysis. The cells were washed with cold PBS, lysed using radioimmunoprecipitation assay (RIPA) buffer (BioWORLD, Dublin, OH, USA) supplemented with protease (Sigma, St. Louis, MO, USA) and phosphatase (Boston BioProducts, Ashland, MA, USA) inhibitor cocktails, incubated on ice for 15–20 min, and then centrifuged at 13,000× *g* for 15 min. The supernatant was collected, and the protein concentration was measured with the Pierce BCA protein assay kit (Thermo Scientific, Rockford, IL, USA) using the TECAN software. Proteins (20 or 60 μg/lane) were separated on a 4–12% SurePAGE Bis-Tris gel (GeneScript, Piscataway, NJ, USA) and transferred for 1 h onto a low-fluorescence polyvinylidene difluoride (PVDF) membrane (Biorad Laboratories, Hercules, CA, USA). Non-specific interactions were blocked using 5% BSA (Sigma, St. Louis, MO, USA) and 0.1% Tween 20 in Tris-buffered saline. The membrane was then immunoblotted overnight at 4 °C with different primary antibodies (1:1000 dilution) directed to hERG1 CT-pan (Enzo Life Science, Lausen, Switzerland), AMPKα, and mTOR Ser2448-specific and total (Cell Signaling Technology, Danvers, MA, USA). β-actin (1:1000, Cell Signaling Technology, Danvers, MA, USA) or vinculin (1:5000, Santa Cruz Biotechnology, Dallas, TX, USA) expression was used as the loading control. The membrane was then probed with anti-rabbit IRDye 680RD and anti-mouse IRDye 800CW secondary antibodies (1:15,000; LI-COR, Lincoln, NE, USA) for 1 h at room temperature, and blot images were obtained using a LI-COR blot scanner. The band density was quantified using the ImageJ software (NIH) and normalized to that for the corresponding housekeeping protein. The relative levels of phosphorylated proteins were further normalized to each respective total protein level, and then normalized to the control. Unless or otherwise specified, cells were pretreated (≥30 min) with test drugs and then subsequently exposed (2 h) to BSA-palmitate (0.5 mM, to induce lipotoxicity) in the continued presence of the specified test compound.

### 4.7. Data Analyses

Electrophysiological data were analyzed offline using built-in functions in Clampfit (pClamp) and the Origin software. The current amplitudes (in pA) were divided by the cell size (in pF) and are expressed as the current densities (pA/pF). The data are reported as the means ± S.E.M. Statistical differences were determined using one-way ANOVA with Bonferroni post hoc analysis or two-tailed unpaired *t* tests for comparisons between groups and considered significant at *p <* 0.05.

## 5. Conclusions

Our methodological chain involved both native guinea pig atrial myocytes and HEK-hERG1a/1b cells, revealing consistent findings. Thus, it is intriguing to speculate that targeted interventions in large animal models of obesity and supraventricular arrhythmias that prevent either (1) mTOR activation (drugs that promote reduced protein synthesis or decrease cardiac or systemic lipid levels) or (2) pathological increases in *I_Kr_* channel function (cellular mediators that reduce channel opening) may be antiarrhythmic and, therefore, beneficial to obese patients that display vulnerability to atrial arrhythmogenesis.

## Data Availability

All the relevant data are included within the paper itself.
